# MRI investigation of the sensorimotor cortex and the corticospinal tract after acute spinal cord injury: a prospective longitudinal study

**DOI:** 10.1016/S1474-4422(13)70146-7

**Published:** 2013-09

**Authors:** Patrick Freund, Nikolaus Weiskopf, John Ashburner, Katharina Wolf, Reto Sutter, Daniel R Altmann, Karl Friston, Alan Thompson, Armin Curt

**Affiliations:** a Spinal Cord Injury Center Balgrist, University Hospital Zurich, University of Zurich, Zurich, Switzerland; b Department of Radiology, University Hospital Balgrist, University of Zurich, Zurich, Switzerland; c Department of Brain Repair and Rehabilitation, UCL Institute of Neurology, University College London, London, UK; d Wellcome Trust Centre for Neuroimaging, UCL Institute of Neurology, University College London, London, UK; e NMR Research Unit, Queen Square Multiple Sclerosis Centre, UCL Institute of Neurology, University College London, London, UK; f Medical Statistics Department, London School of Hygiene and Tropical Medicine, London, UK; g National Institute for Health Research, UCL Hospitals Biomedical Research Centre, London UK

## Abstract

**Background:**

In patients with chronic spinal cord injury, imaging of the spinal cord and brain above the level of the lesion provides evidence of neural degeneration; however, the spatial and temporal patterns of progression and their relation to clinical outcomes are uncertain. New interventions targeting acute spinal cord injury have entered clinical trials but neuroimaging outcomes as responsive markers of treatment have yet to be established. We aimed to use MRI to assess neuronal degeneration above the level of the lesion after acute spinal cord injury.

**Methods:**

In our prospective longitudinal study, we enrolled patients with acute traumatic spinal cord injury and healthy controls. We assessed patients clinically and by MRI at baseline, 2 months, 6 months, and 12 months, and controls by MRI at the same timepoints. We assessed atrophy in white matter in the cranial corticospinal tracts and grey matter in sensorimotor cortices by tensor-based analyses of T1-weighted MRI data. We used cross-sectional spinal cord area measurements to assess atrophy at cervical level C2/C3. We used myelin-sensitive magnetisation transfer (MT) and longitudinal relaxation rate (R1) maps to assess microstructural changes associated with myelin. We also assessed associations between MRI parameters and clinical improvement. All analyses of brain scans done with statistical parametric mapping were corrected for family-wise error.

**Findings:**

Between Sept 17, 2010, and Dec 31, 2012, we recruited 13 patients and 18 controls. In the 12 months from baseline, patients recovered by a mean of 5·27 points per log month (95% CI 1·91–8·63) on the international standards for the neurological classification of spinal cord injury (ISNCSCI) motor score (p=0·002) and by 10·93 points per log month (6·20–15·66) on the spinal cord independence measure (SCIM) score (p<0·0001). Compared with controls, patients showed a rapid decline in cross-sectional spinal cord area (patients declined by 0·46 mm per month compared with a stable cord area in controls; p<0·0001). Patients had faster rates than controls of volume decline of white matter in the cranial corticospinal tracts at the level of the internal capsule (right *Z* score 5·21, p=0·0081; left *Z* score 4·12, p=0·0004) and right cerebral peduncle (*Z* score 3·89, p=0·0302) and of grey matter in the left primary motor cortex (*Z* score 4·23, p=0·041). Volume changes were paralleled by significant reductions of MT and R1 in the same areas and beyond. Improvements in SCIM scores at 12 months were associated with a reduced loss in cross-sectional spinal cord area over 12 months (Pearson's correlation 0·77, p=0·004) and reduced white matter volume of the corticospinal tracts at the level of the right internal capsule (*Z* score 4·30, p=0·0021), the left internal capsule (*Z* score 4·27, p=0·0278), and left cerebral peduncle (*Z* score 4·05, p=0·0316). Improvements in ISNCSCI motor scores were associated with less white matter volume change encompassing the corticospinal tract at the level of the right internal capsule (*Z* score 4·01, p<0·0001).

**Interpretation:**

Extensive upstream atrophic and microstructural changes of corticospinal axons and sensorimotor cortical areas occur in the first months after spinal cord injury, with faster degenerative changes relating to poorer recovery. Structural volumetric and microstructural MRI protocols remote from the site of spinal cord injury could serve as neuroimaging biomarkers in acute spinal cord injury.

**Funding:**

SRH Holding, Swiss National Science Foundation, Clinical Research Priority Program “NeuroRehab” University of Zurich, Wellcome Trust.

## Introduction

Acute trauma to the spinal cord leads to different degrees of sensorimotor and autonomic nerve damage, for which an effective treatment is awaited. Patients with a spinal cord injury generally show little clinical recovery within the first year after injury^1^ and most are permanently disabled. Clinical recovery requires a degree of preservation of axonal tracts and sufficient myelination of fast conducting fibres.^2^ Understanding the sequence of structural and functional changes at the spinal and brain level—and defining their effects on clinical outcome—is key to development of evidence-based rehabilitation therapy.^3^ Moreover, with the advent of clinical trials in this setting,^4^, ^5^ a pressing need exists for in-vivo neuroimaging biomarkers that can reliably assess the extent of neural damage, elucidate the mechanisms of neural repair, and predict clinical outcome.^6^

Study of acute and progressive structural changes in human spinal cord injury is challenging because of the small number of patients with this disorder, the heterogeneity of spinal cord damage, and the difficulty of neuroimaging close to the lesion site in the presence of implants. Although prognostic indicators of neurological and functional recovery^7^ based on gross anatomical changes at the lesion site have been established with conventional MRI,^8^ their use as neuroimaging biomarkers is restricted (eg, because changes in the extent of spinal cord haemorrhage and swelling provide little information about the condition of the nerve fibre tracts).

In chronic spinal cord injury, cross-sectional measures of the spinal cord and brain, such as cord area and voxel-based morphometry (VBM) of cortical volume, have shown correlations between structural changes (ie, atrophy) and neurological deficits.^9^ However, study of cross-sectional samples of patients at a specific timepoint is uninformative for assessment of degenerative (or neuroplastic) processes that manifest over time. These processes are characterised by structural changes at the microstructural (eg, myelination) and macrostructural (volume) level in specific systems and tissues. Characterisation of the pattern of changes that affect the myelin and axonal architecture might be crucial for the design and execution of clinical trials and for most appropriate use of rehabilitation therapies.

On the basis of preclinical work^4^, ^10^ and MRI findings in chronic spinal cord injury,^11^, ^12^, ^13^ we postulated that microstructural and macrostructural changes would follow a specific temporal and spatial pattern after acute spinal cord injury, and the extent of supraspinal morphological changes would be associated with clinical outcomes. Therefore, we used tensor-based morphometry (TBM), applied to longitudinal anatomical MRI data, to assess progression of volumetric changes after injury^14^ and multiparametric mapping (MPM)^15^, ^16^, ^17^ to assess myelination changes as a marker of microstructure.

## Methods

### Participants and study design

In our longitudinal study, we enrolled patients with acute traumatic spinal cord injury who were admitted consecutively to the University Hospital Balgrist (Zurich, Switzerland) between Sept 17, 2010, and Dec 31, 2012. Eligible patients had acute (within the past 2 months) traumatic spinal cord injury, without head or brain lesions associated with the spinal cord injury, pre-existing neurological disorders, medical disorders leading to functional impairment or mental illness, or contraindications to MRI.

Patients underwent a comprehensive clinical assessment, including the international standards for the neurological classification of spinal cord injury (ISNCSCI) protocol^18^ and the spinal cord independence measure (SCIM),^19^ at baseline and at 2 months, 6 months, and 12 months of follow-up. Both scales range from 0 to 100, with 0 indicating no impairment and 100 indicating full impairment. At each timepoint, patients underwent a comprehensive MRI protocol.

We also enrolled healthy individuals from the local area, who were invited to partake in MRI investigations, following the same schedule as the patients. All participants provided written informed consent before participation in the study, which was approved by the local Ethics Committee of Zurich (EK-2010-0271).

### Procedures

We scanned participants with a 3T Magnetom Verio MRI scanner (Siemens Healthcare, Erlangen, Germany) operated with a 16-channel receive head and neck coil. The MRI protocol consisted of assessment of three-dimensional (3D) whole-brain (including brainstem and cervical cord [C1–C5]) structural volume data with an optimised^20^ high-resolution T1-weighted 3D Magnetization Prepared Rapid Acquisition Gradient-Echo (MPRAGE) sequence^21^ at each visit and multiparametric mapping based on multiecho 3D fast low angle shot (FLASH) sequences at 12 months.^22^

For T1-weighted data, 176 sagittal partitions were acquired with an isotropic spatial resolution of 1 mm^3^ in a total scan time of 9·04 min. The imaging parameters were: field of view of 224×256 mm^2^, matrix 224×256, repetition time of 24·20 ms, echo time of 4·18 ms, inversion time of 960 ms, flip angle of 9°, and readout bandwidth of 150 Hz per pixel. We checked all images for movement artifacts. The MPM protocol was based on the Siemens 3D FLASH product sequence with the following sequence parameters:^17^, ^23^ We applied a repetition time of 25 ms and flip angle of 23° to the T1-weighted images, a repetition time of 25 ms and flip angle of 4° for the proton density-weighted images, and a repetition time of 37 ms and flip angle of 9° for the magnetisation transfer (MT)-weighted images. MT weighting was achieved by an off-resonance radiofrequency pulse before non-selective excitation. Alternating gradient echoes were acquired at seven equidistant echo timepoints between 2·46 ms and 17·22 ms for the MT-weighted acquisitions with one additional echo at time 19·68 ms for the T1-weighted and proton density-weighted acquisitions. Total scan time for the three 3D FLASH datasets was 23 min.

We used Jim 6·0 (Xinapse systems, Aldwincle, UK) for the measurement of cross-sectional spinal cord area. First, we extracted a series of ten contiguous reformatted axial slices (3 mm slice thickness) at the C2/C3 level based on a previously published method.^23^ The cross-sectional cord area was then calculated automatically using an active-surface model.^24^

We used TBM, as implemented in SPM12 (University College London, London, UK), to estimate regional changes of brain volume over time for patients and controls.^14^ By use of groupwise-consistent 3D non-linear image registration, we longitudinally aligned the four MRI volumes for each participant, collected at baseline, 2 months, 6 months, and 12 months, to their midpoint average^14^ to produce a Jacobian determinant map for each timepoint—relative to the midpoint average image—for each participant. The average images were subsequently aligned with the population mean, enabling the Jacobian difference maps to be transformed (with Jacobian scaling) to the same standard (Montral Neurological Institute [MNI]) space.^14^ These aligned maps encoded the three-dimensional profiles of longitudinal volumetric expansion and compression for each participant.

We used VBM to make voxel-wise comparisons of white matter and grey matter volume between groups of participants at 12 months.^25^ Briefly, the T1-weighted anatomical images were segmented into grey matter, white matter, and CSF with unified segmentation.^26^ For each participant, this procedure produced three images in the same space as the original anatomical image, in which each voxel was assigned a probability of being grey matter, white matter, or CSF. The grey matter and white matter segments were then warped into standard MNI space, representing the average of all the participants, with a diffeomorphic non-linear image registration instrument implemented in SPM12 (Dartel).^27^ The grey matter volumes were scaled with the Jacobian determinants from the registration step (ie, modulation) to preserve the local tissue volumes. Finally, the volumes were smoothed with an isotropic Gaussian kernel with 5 mm full width at half maximum.

We used MT-weighted, proton density-weighted, and T1-weighted FLASH images to calculate the quantitative parameter maps of MT saturation and apparent longitudinal relaxation rate R1 (defined as 1/T1) as described previously.^17^, ^22^, ^28^ We corrected R1 brain maps for radiofrequency transmit field inhomogeneities with UNICORT.^22^ By use of unified segmentation, the MT maps of each participant were segmented into grey matter, white matter, and CSF^26^ and transformed to standard MNI space—again with Dartel.^27^ Then, we warped the MT and corrected R1 maps to standard MNI space with the participant-specific diffeomorphic estimates from the Dartel procedure and smoothed with an isotropic Gaussian kernel with 5 mm full width at half maximum, while preserving the parameter values with the voxel-based quantification (VBQ) renormalisation.^22^

Because our main focus was on degenerative changes of the sensorimotor system caused by trauma to the spinal cord,^9^ we created a binary mask from the MRI Atlas of Human White Matter^29^ derived from diffusion imaging in MNI space to assess in the normalised images (also in MNI space) the volumetric and microstructural changes encompassing the corticospinal tracts. To assess grey matter changes in the cortex we extracted the bilateral primary motor (M1) and primary sensory (S1) cortices with the anatomy toolbox for SPM.^30^

### Statistical analysis

We used Stata 12 to assess clinical recovery and spinal cord area change. We used regression models to estimate rates of change of spinal cord area (all participants) and clinical recovery (patients only), with the spinal cord area, clinical measure, or lesion level as response variable and time as predictor. For clinical measures, time was modelled on a log scale to accommodate non-linear recovery trajectories. A quadratic term in time was added to model non-linear changes in cord area with time. To compare rates of change of cord area between patients and controls, we included a group indicator and group × time interaction in the regression model. Age and sex terms and their interaction with time were included to accommodate potential age or sex confounds.

To assess longitudinal cortical changes over 12 months, we used statistical parametric mapping to test for regionally specific changes in grey and white matter volume as determined by the TBM Jacobian. These data were modelled with (general linear) regression models comprising group indicators, age, and time. We expected that volume changes would depend non-linearly on time (ie, on the time elapsed between measurement and injury) because saturation and rebound effects might occur. Thus, we modelled the time dependence of the volume with linear and quadratic terms as previously described. The constant term models a general offset (intercept), the linear term models volume changes with a constant rate of atrophy, and the quadratic term models changes with an accelerating and decelerating rate of atrophy.

The model was fitted to every voxel in the regions of interest in the bilateral M1 and S1 cortices for grey matter volume and the cranial corticospinal tracts for white matter volume. We used *t* statistics to test for linear and quadratic volume changes over time. The *t* tests were one-tailed and the associated p values of less than 0·05 were corrected for multiple comparisons with family-wise error correction within each region of interest, with Gaussian random field theory to accommodate non-stationary spatial correlations among error terms.^14^

To assess differences in grey matter and white matter volume and myelination (MT and R1 maps) between patients and controls at 12 months, we used statistical parametric mapping and the T1-weighted 3D MPRAGE for VBM analyses and MPM data for VBQ analyses. Our regression models included group indicators, age, and total intracranial volume. We calculated total intracranial volume from the segmented grey matter, white matter, and CSF from the T1-weighted 3D MPRAGE scans for the VBM analysis and from the MT scans for the VBQ analysis. This analysis used the same regions of interest and statistical thresholds as in the TBM analysis.

To examine associations between spinal cord area and clinical recovery in patients, regressions were done with clinical outcome at 12 months as response and change in cord area at 12 months as predictor. To assess associations between cortical volume changes and clinical recovery in time, we used the linear and quadratic coefficients (ie, parameter estimates from the aforementioned regression models) as response variables and clinical covariates of interest (ISNCSCI motor score, SCIM score) and age as predictor variables to produce statistical parametric maps of regionally specific associations. These associations were tested with *F* statistics and were regarded as significant if p<0·05 (corrected for multiple comparisons as described previously).

### Role of the funding source

The sponsor of the study had no role in study design, data collection, data analysis, data interpretation, or writing of the report. All authors had full access to all the data in the study and responsibility for the decision to submit for publication.

## Results

We recruited 13 patients with acute traumatic spinal cord injury (12 men and one woman), with a mean age of 46·9 years (SD 20·2; table 1). We recruited 18 healthy participants (12 men and six women; mean age 35·0 years [9·3]).

**Table 1:** Clinical and behavioural data for 13 patients with spinal cord injury

	**Age at injury (years)**	**Injury**		**ISNCSCI grade at baseline**	**Initial site of impairment (motor/sensory)**	**ISNCSCI motor score (maximum 100)**		**SCIM score (maximum 100)**	
		Type	Severity			Baseline	12 months	Baseline	12 months
1	19	Fall	Complete	A	C5/C4	20	23	4	27
2	23	Fall	Incomplete	B	C7/C6	42	67	23	70
3	70	Fall	Incomplete	B	T10/T10	62	75	38	42
4	75	Fall	Incomplete	D	T12/T12	100	NA*	60	NA*
5	44	Fall	Incomplete	D	T11/T11	89	95	39	100
6	42	Fall	Complete	A	C5/C5	23	25	1	37
7	71	Fall	Incomplete	B	C7/C8	52	89	17	36
8	20	MVA	Complete	A	C5/C5	21	23	4	34
9	30	MVA	Incomplete	B	C7/C8	22	48	13	38
10	52	Fall	Incomplete	D	T9/T9	98	98	47	100
11	42	MVA	Incomplete	D	C5/C4	81	82	98	98
12	70	MVA	Complete	A	T7/T7	50	50	29	45
13	52	MVA	Incomplete	B	C6/C6	27	44	24	36

All patients were male, apart from patient 3 who was female. ISNCSCI=international standards for the neurological classification of spinal cord injury. SCIM=spinal cord independence measure. MVA=motor vehicle accident.

* Not available because the patient died.

Eight of 13 patients with a traumatic spinal cord injury had tetraplegia and five had paraplegia (table 1). Three patients with tetraplegia and one patient with paraplegia were classified as having complete spinal cord injury based on the ISNCSCI classification.

From time of injury, the mean interval to the baseline scan was 35·48 days (SD 17·94), to the 2 month scan was 77·08 days (16·88), to the 6 month scan was 177·75 days (24·81), and to the 12 month scan was 356·75 days (34·22). We included 31 datasets at baseline and 30 datasets at 2 months, 6 months, and 12 months in the final analysis, meaning 30 (98%) of 31 participants completed follow-up. One patient missed the second scan owing to illness and another patient died 5 months after enrolment from a cause unrelated to spinal cord injury.

In the 12 months from baseline, patients recovered by 5·27 points per log month (95% CI 1·91–8·63) on the ISNCSCI motor scores scale (p=0·002) and by 10·93 points per log month (6·20–15·66) on the SCIM score scale (p<0·0001). Approximate linearity of recovery on the log time scale implies that the recovery rate is faster soon after the injury than it is later on. In particular, when ISNCSCI and SCIM score recovery were modelled on both raw time and log time as covariates, the raw time coefficient lost significance (p=0·061 for ISNCSCI and p=0·137 for SCIM) whereas log time remained significant (p=0·0010 and p=0·0001), suggesting that log time fits the recovery trajectory better than did raw time, and therefore that recovery is steeper in the first 6 months than it is after this point.

**Figure 1:**
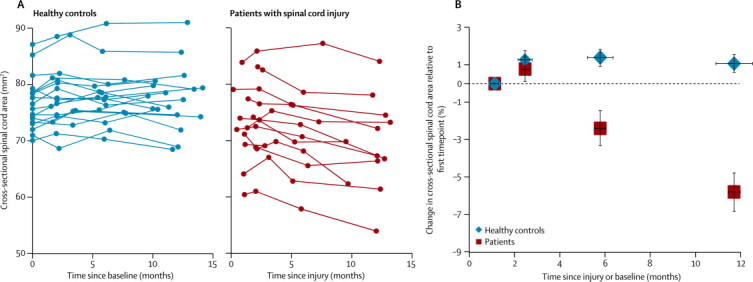
Longitudinal changes in spinal cord area (A) Change in cross-sectional spinal cord area at the C2/C3 level after injury in patients with spinal cord injury and in healthy controls. (B) Change in cross-sectional spinal cord area differed significantly between patients and controls. Horizontal error bars show SE of the scan intervals and vertical error bars show SE for percentage change in cross-sectional spinal cord area.

At baseline, spinal cord area was 3·7 mm^2^ lower in patients with a spinal cord injury (mean 73·0 mm^2^ [SD 6·8]) than it was in controls (mean 76·7 mm^2^ [4·6]; p=0·089). Patients had a significantly greater rate of change of spinal cord area than did controls (patients decreasing by 0·46 mm^2^ per month [95% CI 0·58–0·33] more than controls, p<0·0001; figure 1). In patients, spinal cord area decreased by 0·41 mm^2^ per month (0·51–0·31, p<0·0001), whereas in controls the spinal cord area did not change substantially (increase of 0·048 mm^2^ per month −0·03 to 0·13, p=0·234; figure 1). We did not detect any significant non-linear (quadratic) spinal cord atrophy (p=0·718).

**Figure 2:**
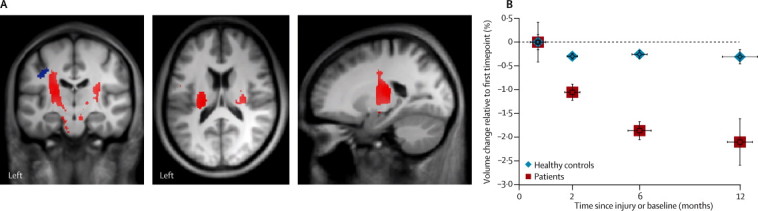
Longitudinal changes in grey and white matter volume shown by tensor-based morphometry (A) Overlay of statistical parametric maps (uncorrected p<0·001, shown for descriptive purposes, masked by the union of the cranial corticospinal tract and the bilateral sensorimotor cortex) showing regions of volume changes in grey matter (in blue) and white matter (in red). (B) Illustration showing changes in white matter volume in the corticospinal tracts, at the level of the left internal capsule, in patients and healthy controls. Horizontal error bars show SE of the scan intervals and vertical error bars show SE for percentage change in spinal cord area.

At the cortical level, patients showed a significant difference in rates of change of white matter volume in the cranial corticospinal tracts (figure 2). These effects were noted at the level of the internal capsule (right side *Z* score 5·21, p=0·0081; left side *Z* score 4·12, p=0·0004) and the right cerebral peduncle (*Z* score 3·89, p=0·0302). The rate of atrophy decelerated (with a significant quadratic effect) within the left internal capsule between 6 months and 12 months follow-up (*Z* score 4·88, p=0·0265; figure 2, table 2). Rates of cortical grey matter volume changes differed between patients and controls in the left primary motor cortex (*Z* score 4·23, p=0·041). We did not detect any significant non-linear (quadratic) effects for grey-matter changes.

**Table 2:** Progressive white and grey matter atrophy in patients with spinal cord injury

	***Z* score**	**p value (FWE-corrected)**	**Cluster extent (voxels)**	***x* (mm)**	***y* (mm)**	***z* (mm)**
**Linear rate of atrophy**						
Right internal capsule	5·21	0·0081	2180	−19·5	−22·5	16·5
Left internal capsule	4·12	0·0004	1074	22·5	−25·5	12·0
Right cerebral peduncle	3·89	0·0302	56	−9·0	−15·0	−22·5
Left primary motor cortex	4·23	0·0410	442	−43·5	−13·5	39·0
**Deceleration of rate of atrophy**						
Left internal capsule	4·88	0·0265	1094	−19·5	−22·5	16·5

Only clusters with significant rates of atrophy between patients and controls are shown for the tensor-based morphometry analysis. FWE=family wise error.

**Figure 3:**
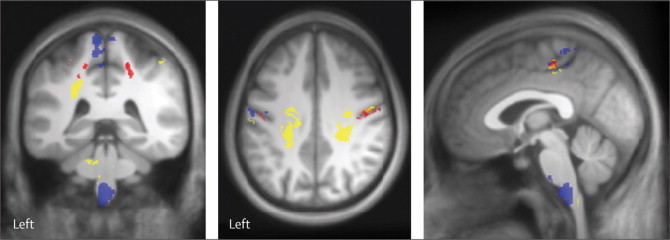
Changes at 12 months shown by voxel-wise analysis of microstructure and volume Overlay of statistical parametric maps (uncorrected p<0·001, shown for descriptive purposes, masked by the union of the cranial corticospinal tract and the bilateral sensorimotor cortex) showing regions of reduced volume of grey and white matter (blue) in patients compared with controls at 12 month follow-up. The reductions in longitudinal relaxation rate R1 (yellow) and magnetisation transfer MT (red) in patients compared with controls at 12 month follow-up suggest changes in myelination.

Figure 3 shows the reduction in cortical grey matter volume and subcortical white matter volume in the corticospinal tracts at the level of the medulla oblongata at 12 months in patients relative to controls. This reduction was paralleled by reductions of R1 and MT—which are myelin sensitive—within hand and leg areas of primary motor and primary sensory cortices and the corticospinal tracts. The reduction in R1 and MT overlapped with areas of the sensorimotor cortex and corticospinal tracts that showed progressive atrophy (table 3).

**Table 3:** Group analyses showing cortical changes both of atrophy and microstructure in patients compared with controls at 12 months

			***Z* score**	**p value (FWE-corrected)**	**Cluster extent (voxels)**	***x* (mm)**	***y* (mm)**	***z* (mm)**
**M1 or S1 region of interest**								
Left								
	R1							
		M1	4·04	<0·001	169	−1·0	−24·0	56·0
		M1	3·48	0·0469	30	−44·0	−13·0	43·0
		S1	4·12	0·0009	45	−52·0	−16·0	46·0
		S1	3·76	0·001	54	−58·0	−19·0	41·0
		S1	4·09	0·0061	34	−32·0	−35·0	52·0
		S1	3·84	0·0201	29	−33·0	−43·0	68·0
	MT							
		M1	5·03	<0·001	89	−1·0	−24·0	54·0
		M1	4·15	<0·001	168	−37·0	−17·0	43·0
		M1	3·92	<0·001	96	−30·0	−25·0	54·0
		S1	3·92	<0·001	310	−45·0	−17·0	35·0
		S1	3·99	0·0027	38	−32·0	−35·0	52·0
		S1	4·40	<0·001	66	−29·0	−41·0	56·0
	VBM							
		M1	4·11	0·0398	235	−7·5	−37·5	73·5
Right								
	R1							
		M1	3·86	<0·001	137	50·0	−6·0	38·0
		S1	4·19	<0·001	105	35·0	−41·0	56·0
		S1	4·48	<0·001	139	55·0	−14·0	40·0
		S1	3·95	0·0007	34	8·0	−32·0	78·0
		S1	4·08	0·0026	46	46·0	−36·0	54·0
		S1	4·02	0·0042	38	9·0	−46·0	76·0
		S1	4·27	0·0124	44	59·0	−19·0	47·0
	MT							
		M1	4·91	<0·001	164	3·0	−29·0	61·0
		M1	4·85	<0·001	85	27·0	−28·0	59·0
		M1	4·84	<0·001	76	34·0	−35·0	54·0
		M1	4·73	<0·001	104	24·0	−28·0	58·0
		S1	5·40	<0·001	819	48·0	−11·0	34·0
		S1	4·73	<0·001	71	59·0	−6·0	29·0
		S1	4·17	0·0304	34	59·0	−19·0	47·0
**CST region of interest**								
Left								
	R1		3·88	<0·001	4033	−32·0	−34·0	28·0
	VBM		4·49	0·0248	203	−6·0	−33	−58·5
Right								
	R1 (cluster 1)		3·58	<0·001	68	11·0	−43·0	−33·0
	R1 (cluster 2)		3·41	0·0035	107	6·0	−43·0	−52·0
	MT		4·10	0·0083	322	20·0	−40·0	51·0

Only clusters with significant differences between patients and controls are shown for the VBM and MPM data (ie, MT and R1). FWE=family wise error. VBM=voxel-based morphometry. CST=corticospinal tract. MPM=multiparametric mapping. MT=magnetisation transfer. R1=longitudinal relaxation rate. M1=primary motor cortex. S1=primary sensory cortex.

**Figure 4:**
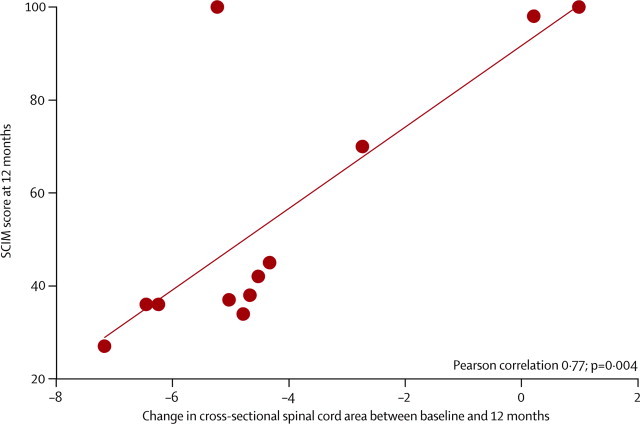
Correlation between spinal cord atrophy and clinical outcome SCIM=spinal cord independence measure.

**Figure 5:**
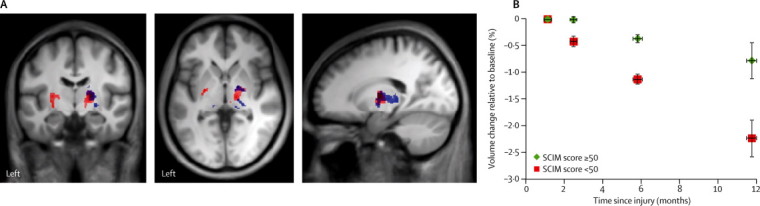
Correlation between brain atrophy and clinical outcome (A) Overlay of statistical parametric maps (uncorrected p<0·001, shown for descriptive purposes; masked by the union of the cranial corticospinal tract) showing associations of volume changes with ISNCSCI motor score (blue) and SCIM score (red). (B) Rates of change in atrophy in patients with a SCIM score <50 and ≥50 at 12 months. Horizontal error bars show SE of the scan intervals and vertical error bars show SE for percentage change in white matter volume change of the corticospinal tract at the level of the internal capsule relative to baseline. SCIM=spinal cord independence measure. ISNCSCI=international standards for the neurological classification of spinal cord injury.

Within the spinal cord, a smaller loss in area during 12 months' follow-up was associated with better SCIM score at 12 months (figure 4). Within the brain, a smaller loss in corticospinal tract white matter volume over time was associated with better outcome at 12 months. Specifically, improved ISNCSCI motor scores (*Z* score 4·01, p<0·0001) and SCIM scores (*Z* score 4·30, p=0·0021) at 12 months were associated with decreased white matter volume changes at the level of the right internal capsule. In addition, an improved SCIM score was associated with decreased white matter volume change encompassing the corticospinal tracts at the level of the left internal capsule (*Z* score 4·27, p=0·0278) and left cerebral peduncle (*Z* score 4·05, p=0·0316; figure 5, table 4). Lesion level was not associated with rate of atrophy (p=0·459).

**Table 4:** Structural correlates of clinical outcome at 12 months

		***Z* score**	**p value (FWE-corrected)**	**Cluster extent (voxels)**	***x* (mm)**	***y* (mm)**	***z* (mm)**
**SCIM score**							
Right internal capsule		4·30	0·0021	539	18·0	−9·0	7·5
Left internal capsule							
	Cluster 1	4·27	0·0278	200	−27·0	−9·0	−1·5
	Cluster 2	4·05	0·0439	57	−7·5	−15·0	−7·5
Left cerebral peduncle		4·05	0·0316	46	−9·0	−31·5	−22·5
**ISNCSCI motor score**							
Right internal capsule		4·01	<0·001	725	16·5	−4·5	−4·5

Only clusters showing significant associations between clinical outcome and volumetric structural changes with time are shown. FWE=family-wise error. SCIM=spinal cord independence measure. ISNCSCI=international standards for the neurological classification of spinal cord injury.

## Discussion

Our study showed that, in patients with acute spinal cord injury, a progressive and specific pattern of structural neural change occurs rostral to the level of the lesion, akin to that reported in studies of animals (panel). As early as 40 days, a pronounced reduction of spinal cord area occurs compared with controls, with a corresponding reduction in white matter volume of the cranial corticospinal tracts and grey matter volume in the sensorimotor cortex. Assessment of the underlying pathophysiology, with a myelin-sensitive MPM approach, supports the assumption that these volume changes relate to atrophy of myelinated axons and their cell bodies within grey matter of the sensorimotor cortices.^11^, ^12^, ^13^ Crucially, from a clinical perspective, a notable association exists between increased changes in spinal cord and brain structure and poor recovery. The finding of a systematic degenerative pattern with time suggests that non-invasive MRI measures could be used for prediction of outcome, identification of patients most likely to benefit from different interventions, and as potential markers of treatment effects of interventions (physical, drug, or cell-based therapies).

Panel **Research in context**

**Systematic review**

We searched PubMed for articles published in English between Jan 1, 1990, and Feb 28, 2013, with the search term “spinal-cord injury” combined with specific search terms that constituted the subheadings (eg, “acute”, “atrophy”, “MRI”, “rehabilitation”, “degeneration”, “functional recovery”). This search identified studies, ranging from changes at the molecular level in animal models of spinal cord injury to changes in macrostructure in people with spinal cord injury. In studies that are most closely related to this study, cross-sectional volumetric changes were assessed in the chronic phase^11^, ^12^, ^13^ but not longitudinally.

**Interpretation**

Previously, degenerative changes of the CNS, remote from spinal cord injury, were thought to occur slowly^31^, ^32^ and relate to the degree of impairment. By use of repeat MRI scans, we showed that these structural changes occur early and progress with a specific spatial and temporal pattern at the spinal and brain levels. These structural changes are assumed to be induced by retrograde degeneration of central motor nerve fibres along the myelinated corticospinal tract axons that over time results in shrinkage of the soma of corticospinal projecting neurons.^32^ Preservation of structural integrity, at the spinal and brain levels, was associated with improved neurological and functional outcomes during rehabilitation. These structural changes might be useful in reporting the effects of interventions targeting neural repair and plasticity, and for optimisation of rehabilitation programmes. Therefore, if confirmed in other studies, structural volumetric and microstructural MRI protocols remote from the site of spinal cord injury could be considered as neuroimaging biomarkers for interventional studies, because they disclose spatially and temporally distinct neuropathological changes in the CNS, and complement clinical outcome measures during rehabilitation from acute spinal cord injury.

Spinal cord and brain areas showed distinct temporal profiles of changes in response to acute spinal cord injury. Spinal cord area decreased consistently by about 7% (5 mm^2^) within the first year after injury. Because in the chronic disease state (14 years after injury) spinal cord area was shown to only be decreased by up to 30%,^12^ an ongoing but slower decrease was to be expected in our patients. As with spinal cord area, corticospinal tract white matter volume showed a progressive decrease (>2% compared with controls) during the same period. By contrast with the spinal cord area, the rate of brain atrophy was non-linear with a marked deceleration after 6 months. Crucially, myelin-sensitive MRI parameters^15^, ^16^, ^17^ at 12 months (R1 and MT) were reduced within, but also beyond, atrophic areas. These findings are suggestive of retrograde degeneration of myelinated axons.^10^ Moreover, the grey matter volume in the leg area of primary motor cortex and white matter volume of the corticospinal tracts (at the level of the pyramids) were decreased to a similar extent as that observed in the chronic phase.^12^ The progressive pattern of spinal cord area and cranial corticospinal tract volume decrease within the first 40 days corresponds to the time course of atrophy of myelinated axons undergoing retrograde degeneration—as described in post-mortem human spinal cord injury tissue^33^ and animal models of spinal cord injury.^10^ The rate of spinal cord atrophy was unrelated to the level of the lesion, suggesting distant independent degenerative processes induced by trauma. Future longitudinal studies combining high resolution quantitative and functional neuroimaging might provide further insights into the mechanisms of degeneration and plasticity and their effect on cortical reorganisation during the course of recovery and rehabilitation.

Although imaging measures have been recognised as the most valuable in-vivo proxies for pathological changes in other neurological diseases, such as multiple sclerosis,^6^ they are not well established in spinal cord injury. The paucity of MRI measures in spinal cord injury is, in part, due to the difficulty in assessment of changes at the lesion level, because of artifacts caused by implanted fixative devices. Therefore, we applied advanced MRI measures that have been used successfully in the field of multiple sclerosis to detect remote structural changes sensitively and accurately. Notably, we show progressive structural changes that were associated with neurological (ISNCSCI) and functional (SCIM) improvement. Specifically, at 12 months, reduced atrophy of the spinal cord was associated with improved SCIM scores. In addition, patients with low volume change in white matter in the cranial corticospinal tract at the level of the internal capsule had improved outcomes on ISNCSCI motor and SCIM scores. In other words, patients with greater corticospinal tract integrity recovered greater muscle strength and had higher degrees of independence (by means of increased ISNCSCI motor and SCIM scores) than did patients with low corticospinal tract integrity. Notably, dynamic atrophic changes at the level of the internal capsule were associated with clinical scores in contrast to those of the final (ie, 12 months) decrease of white matter volume at the level of the medulla oblongata. This effect might be due to statistical effects (statistical power in a cross-sectional versus longitudinal sample) or to the fact that changes in volume at 12 months represent late stage effects that are less sensitive to disease progression than dynamic readouts. These findings might be of value to advance the stratification of patients for selection of drug or rehabilitation strategies and to assess treatment effects by comparing functional outcomes with morphometric changes.^34^ The opportunity to assess the effectiveness of therapeutic interventions (drug or physical therapy) due to insights into dynamic degenerative processes might provide important guidance to optimise rehabilitation strategies.

Our study had some limitations. Only 13 patients were included in the study, because these were the only patients within the recruitment period who fulfilled the rigorous inclusion criteria, although this number should be considered in the context of the enormous burden of the disease (in view of the acuteness and severity of the disorder). Despite this limitation, we showed robust changes because the study benefited from a high compliance (98% completed follow-up) and methods that analysed all available datapoints over time. The fact that we noted significant effects suggests that the underlying effect sizes are large. Another limitation was that controls were an average of 12 years younger than were patients. However, we included age as a covariate in all the statistical models to exclude effects related to age. In accordance with previously published work,^35^ we did not note any evidence in this sample that spinal cord area was associated with age. In this cohort, atrophy rates in the spinal cord and brain were not significantly associated with age, and estimates of rates of change were not materially altered by adjustment for this variable. Finally, our inferences about the underlying disease mechanisms need to be treated with caution, because VBQ and VBM are indirect markers and cannot directly probe underlying biological changes at the microscopic level. However, histopathological studies have revealed a strong association between changes in myelination and MT and R1 values^36^ and therefore changes in myelinated axons can be reasonably assumed to contribute to atrophic changes observed within the first year after spinal cord injury.

To our knowledge, this is the first in-vivo study to disclose acute and progressive structural changes along the neuroaxis after trauma to the spinal cord in human beings. Notably, decreased rates of atrophy were associated with improved clinical outcome. Thus, neuroimaging protocols can reliably capture progressive structural changes at the microstructural and macrostructural levels, and these changes are associated with clinical improvement. Their high sensitivity to change and clinical outcome might support their use as neuroimaging biomarkers to help develop evidence-based rehabilitation therapy and to track changes induced by treatment in trials promoting spinal cord repair.

**This online publication has been corrected. The corrected version first appeared at thelancet.com/neurology on July 16, 2013**
